# Diversity of Chagas disease diagnostic antigens: Successes and limitations

**DOI:** 10.1371/journal.pntd.0012512

**Published:** 2024-10-01

**Authors:** Tapan Bhattacharyya, Niamh Murphy, Michael A. Miles

**Affiliations:** Faculty of Infectious and Tropical Diseases, London School of Hygiene & Tropical Medicine, Keppel Street, London, United Kingdom; FDA: US Food and Drug Administration, UNITED STATES OF AMERICA

## Abstract

Chagas disease, caused by the protozoan parasite *Trypanosoma cruzi*, remains a public health issue in endemic regions of the Americas, and is becoming globalised due to migration. In the chronic phase, 2 accordant serological tests are required for diagnosis. In addition to “in-house” assays, commercial tests are available (principally ELISA and rapid diagnostic tests). Herein, we discuss the discovery era of defined *T*. *cruzi* serological antigens and their utilisation in commercialised tests. A striking feature is the re-discovery of the same antigens from independent studies, and their overlapping use among commonly reported commercial serological tests. We also consider reports of geographical variation in assay sensitivity and areas for refinement including applications to congenital diagnosis, treatment monitoring, and lineage-specific antigens.

## Introduction

In 1907, the physician Carlos Chagas working on an anti-malaria campaign in Minas Gerais state, central Brazil, noticed domestic hematophagous insects carrying flagellated protozoa; primates experimentally infected by these insects developed circulating trypanosomes. Chagas recognised identical parasites in the severe human infections in Minas Gerais. Thus a novel pathogen, *Trypanosoma cruzi*, its triatomine bug vector and associated human disease, named after its founding discoverer, were revealed [1].

Subsequently, *T*. *cruzi* and Chagas disease were recognised throughout the Americas, although only a minority of the approximately 125 triatomine bugs species have been identified as vectors for human infection. Despite control programmes leading to regional decreases in vector distribution, Chagas disease endures as an important public health issue in the Americas, with an estimated 6 million cases [2]. Factors include congenital and oral transmission routes, the chronic nature of apparently asymptomatic infection, and deficiencies in appropriate and timely diagnosis. This can be exacerbated in populations that are more difficult to access by health professionals. The role of domestic and sylvatic animals in the ecology of *T*. *cruzi*, originally also described by Chagas, creates an environmental reservoir for this parasite [3].

Although considered a single species, diversity among *T*. *cruzi* isolates had been long recognised, leading to the identification of disparate enzymic “strain-groups” in domestic and silvatic infections [4,5]. Subsequent analysis of these zymodemes based on random amplified polymorphic DNA (RAPD) and PCR resulted in classification into 6 “discrete-typing units” (DTUs) [6,7] later named TcI-TcVI [8], and also TcBat [9]. The genetic targets were non-antigen coding. TcV and TcVI are natural hybrids [10]; the existence of hybridisation mechanisms has been demonstrated experimentally [11,12]. Messenger and colleagues [13] present a comprehensive history of nomenclatures used to classify *T*. *cruzi*.

In the acute phase of human infection, specific diagnosis can be achieved by demonstration of circulating *T*. *cruzi*. During the chronic phase, in which approximately 30% of people may exhibit cardiac pathology and/or gastrointestinal megasyndromes, serology (IgG detection) becomes more relevant. WHO recommends that at least 2 accordant serological tests are used for diagnosis in the chronic phase [14]. However, there is a wide range of serological tests and formats, including commercialised and “in-house” assays using defined antigen sequences or an undefined mix derived from cultures.

In this article, we present a survey of the defined *T*. *cruzi* antigens that have been utilised in commercialised diagnostic tests for Chagas disease. We consider their discovery, application in assay formats, reports of geographical variation in test sensitivity, and opportunities for further research.

## The discovery era of defined antigens

The development of molecular biology techniques during the 1980s was crucial to the improved characterisation of potential serological antigens. In particular, the immuno-screening of *T*. *cruzi* expression libraries cloned into bacteriophage lambda (λ) vectors with sera from humans or experimentally infected animals. The discovery era for key defined antigens as later used in commercialised tests is chronicled in Table 1, in which the TcI-TcVI classification has been applied retrospectively [15]. Of particular importance were the landmark publications of Ibañez and colleagues [16,17], which characterised several antigenic sequences—herein called **Ag1**, **Ag2**, etc.—by screening a library from an isolate derived from an asymptomatic chronic chagasic patient [18]. Remarkably, the same or highly similar sequences were later re-identified (and re-named) from subsequent independent screening strategies using disparate sources of chronic chagasic sera [19–28], and in one case preferentially by acute serum [29], as described in early reviews [30,31]. In Table 1, GenBank accession numbers (where available) relate to these early reports.

**Table 1:** Key *Trypanosoma cruzi* serological antigens identified during the discovery era (1985–2001).

Year	Reference	Antigen (reported function/localization)	*T*. *cruzi* strain (Tc lineage)	Human sera^a^	Experimental sera	Sequence homologies	Genbank^b^
1985	Gonzalez et al. [32]	**1F8**	Y (TcII)	-	-	-	X02838
1987, 1988	Ibañez et al. [16,17]	**Ag1, -2, -7, -13,** **-30, -36**	Miranda/76 (TcI)	ARG, BRA, CHI.	rabbit	-	Sequences given in [17] and M21330 (Ag1); M21582 (Ag7/SAPA); M21331 (Ag36)
1988, 1989	Kirchoff et al. [33], Engman et al. [34]	**FCaBP** (Flagellar calcium-binding protein)	Sylvio X-10/4 cDNA (TcI)	-	murine	**1F8**	L26971
1989	Lafaille et al. [19]	**FRA** (flagellar repetitive antigen) **CRA** (cytoplasmic repetitive antigen)	Dm28c (TcI)	-	rabbit	**Ag1** (FRA); **Ag30** (CRA)	L09564 (FRA); J04016 (CRA)
1989	Levin et al. [20]	**JL7**, -**9**	Tulahuen 2 epimastigote cDNA (TcVI)	ARG	-	**Ag1** (JL7); **Ag36** (JL9)	Sequences given in [20]
1989	Affranchino et al. [29]	**SAPA** (shed acute phase antigen; trans-sialidase C-terminal repeat region	Miranda/76 (TcI)	BRA	-	**Ag7**	M21582
1989	Hoft et al. [21]	**TCR27, -39,** -**69**	Sylvio X-10/4 (TcI)	BOL, SAL	rabbit	**Ag30** (TCR27); **Ag2** (TCR39)	Sequences given in [21]
1991	Kerner et al. [22]	**MAP** (microtubule associated protein)	Tulahuen 2 epimastigote cDNA (TcVI)	-	murine	**Ag36**; **JL9**	AF158722
1992	Burns et al. [23]	**TcD** (trypomastigote protein)	Tulahuen (TcVI)	BRA, BOL, ARG	-	**Ag13**	M82834
1992	Buschiazzo et al. [27]	**CA-2**	CA-1/65 (TcI)	-		**Ag2**	M92049
1993	Gruber and Zingales [24]	**B13** (trypomastigote surface membrane)	Y (TcII)	-	rabbit	**Ag2**; **TCR39**	AY325808
1990, 1995	Cotrim et al. [25, 26]	**H49** (flagellum attachment region)	G (TcI)	BRA	-	**Ag1**; **JL7**; **FRA**	U16294
1996	Porcel et al. [28].	**F29** (calcium-binding protein)	Miranda/76 (TcI)	-	rabbit	**1F8**; **FCaBP**	Z54193
1997	Lesénéchal et al. [35]	**Tc40**	G (TcI)	BRA	-	**TCR69**	U24190
1999	Houghton et al. [36]	**TcE**, **TcLo1.2**, **TcF**	Not stated	BRA, ECU, USA		**TCR69**; **T40** (TcE); TcF is fusion of **Ag2** / **TcD** (**Ag13**) / **TcE** (**TCR69**)	Sequences given in [36]
2000, 2001	Thomas et al. [38, 39]	**KMP-11** (kinetoplastid membrane protein)	Y epimastigote cDNA (TcII)	COL, VEN		-	AJ000077

^a^ ARG, Argentina; BOL, Bolivia; BRA, Brazil; CHI, Chile; COL, Colombia; ECU, Ecuador; SAL, El Salvador; VEN, Venezuela; USA, originating from endemic regions.

^b^ GenBank accessions refer to original or early submissions.

Table 2 shows amino acid conservation between re-identified antigens; for example, Ag1 and JL7/H49/FRA; 1F8 [32] and FCaBP [33,34]; TCR69 [21] within Tc40 [35] and TcE [36]. In cases where the GenBank or original published sequence contains perfect or near-perfect repeats, alternate residues are depicted in Table 2. Shed Acute Phase Antigen (SAPA/Ag7) was later identified as the C-terminal repeat region domain of trans-sialidase involved in host cell invasion [37]. Repetition of sequence may aid in serology by presenting multiple epitopes.

Kinetoplastid membrane protein 11 (KMP-11), identified genomically by screening with the corresponding *Leishmania tropica* region [38,39], does not seem to be a re-identification.

Table 2 also maps the antigen sequences, as identified in the discovery era, to the subsequent CL Brener reference genome accessed via the TriTrypDB (https://tritrypdb.org).

**Table 2:** Discovery era *Trypanosoma cruzi* antigen sequences mapped to CL Brener genome. Sequences are aligned to original report; alternate residues are represented in super- or subscript.

Antigen (year)	Alignment of reported sequences
Ag1 (1988)	------------------------- SMNARAQELAREKKLADRAFLDQKPEGVPLRELPLDDDSDFVAMEQERRQQLEKDPRRNAKREIAAL
FRA (1989)	MEQERRQLLEKDPRRNAR REIAALEE..........................V....DLV..........
JL7 (1989)	-------------...A^R^_K._....................^L^_F_...^E^_A_...............................L.....
H49 (1995)	..............K.VQK..D......VC.RN..F.IRSRE.D.L.DVVR.I..DA...N..NELCLL.SR..DV.KSLQKIILS
Genome	Cysteine peptidase; TcCLB.511441.10
Ag2 (1988)	GD^K^_R_P ^P^_S_^P^_L_FGQ ^G^_A_^A^_T_^A^_V_
TCR39 (1989)	.^D^_E_K. ^S^_P_^P^_L_... A A A
CA-2 (1992)	..K. ^P^_S_^P^_L_... A A A
B13 (1993)	^G^_A_D.^P^_L_^P^_S_^P^_L_..... ^E^_A_
Genome	Nucleoporin; TcCLB.508831.140
Ag7/SAPA (1988)	DS^S^_T_AH^G^_S_TPSTP^V^_A_
Genome	Trans-sialidase, Group I, putative; TcCLB.509495.30
Ag13 (1988)	K^A^_S_^G^_A_EP
TcD (1992)	K^P^_S_A^E^_G_^P^_S_
Genome	Trans-sialidase, Group IV, putative; TcCLB.510307.284
Ag30 (1988)	EKQ^R^_K_AAEATKVAEA
TCR27 (1989)	...^R^_K_........^E^_G_^A^_D_
CRA (1989)	...^R^_K_...^A^_S_1.^V^_A_^V^_A_.^A^_T_
Genome	R27-2 protein, putative; TcCLB.506811.160
Ag36 (1988)	ALPQ^E^_V_EQEDVGPRHVDPDHFRSTT^Q^_H_DAYRPVDPSAYKR
JL9 (1989)	....E.^E^_Q_.................Q.............
MAP (1991)	^A^_T_...E.E.................^Q^_H_.............A
Genome	Microtubule-associated protein; TcCLB.511633.79
1F8 (1985)	MGACGSKGSTSDKGLASDKDGKKAKDRKEAWERIRQAIPREKTAEAKQRRIELFKKFDKNETGKLCYDEVHSGCLEVLKLDEFTPRVRDITK
FCaBP (1988)	-.....................N...............................................Y.............S.......
F29 (1996)	-.....................N...............................................Y.....................
1F8	RAFDKARALGSKLENKGSEDFVEFLEFRLMLCYIYDFFELTVMFDEIDASGNMLVDEEELKRAVPKLEAWGAKVEDPAALFKELDKNGTGSV
FCaBP	.....S.T...................................................F................................
F29	.......V...................................................F................................
1F8	TFDEFAAWASAVKLDADGDPDNVPESA
FCaBP	...........................
F29	.........................K.
Genome	Flagellar calcium-binding 24 kDa protein; TcCLB.509391.30
TCR69 (1989)	AAPAKAA
Tc40 (1997)	S......
TcE (1999)	2......
Genome	60S ribosomal protein L19; TcCLB.509149.60
KMP-11 (2000)	MATTLEEFSAKLDRLDAEFAKKMEEQNKKFFADKPDESTLSPEMKEHYEKFEKMIQEHTDKFNKKMHEHSEHFKAKFAELLEQQKNAQFPGK
Genome	Kinetoplastid membrane protein 11; TcCLB.510755.89
TcLo1.2 (1999)	GTSEEGSRGGSSMP^S^_A_
Genome	Trans-sialidase, putative; TcCLB.499233.10

1, T/M/A; 2, A/T/I

## Application in commercialised diagnostic tests

Prior to the use of defined antigens (i.e., of known sequence), serological tests for *T*. *cruzi* infection were based on whole cell or crude (undefined) preparations, with the potential for confounding cross-reaction with related and co-endemic pathogens such as *Leishmania*. From the 2000s, the exploitation of the defined antigens as used in commercial diagnostic tests in research and clinical contexts, in endemic and non-endemic settings, has taken the principal formats of rapid diagnostic tests (RDTs) and ELISA kits.

A striking feature during the development of different commercial assays from different manufacturers is that they share a high degree of overlap of antigens/epitopes, often listed in manufacturer literature under the varying nomenclatures by which the antigens were re-identified during the discovery era. These commercial tests are listed in Table 3 and further described here:

*RDTs*

Chagas Stat-Pak (https://chembio.com/products/chagas-stat-pak-assay-europe/). In 2003, Luquetti and colleagues [41] reported the development of Chagas Stat-Pak as a novel, immunochromatographic, lateral flow rapid test, utilising a mixture of recombinant B13, 1F8 and H49/JL7 produced separately as GST-fusions in *E*. *coli*. Its use with serum samples from Argentina, Bolivia, Brazil, Honduras, and Venezuela gave comparable results. This RDT has been often reported, including evaluation in a WHO-coordinated multicentric trial of commercial RDTs [42] and in non-endemic settings [43].

SD BIOLINE Chagas Ab Rapid (https://maxanim.com/rapid-tests/sd-bioline-chagas-ab-rapid/) also utilises recombinant antigens H49 and 1F8 [44].

Chagas Detect Plus (https://inbios.com/chagas-detecttm-plus-rapid-test-usa-2/) utilises an *E*.*coli*-produced recombinant SUMO-tagged multi-epitope fusion protein incorporating Ag2-TcD-TcE-SAPA-Ag30-Ag36-Kmp11-Kmp11-Ag1, designated ITC8.2 [40], manufactured as prototype Trypanosoma Detect (InBios, USA) and used in publications under this name [42,45]. Re-named as Chagas Detect Plus, this RDT has been used in endemic [46,47], and non-endemic settings [48]. The recombinant antigen incorporates a mixture of either epitopes or longer sequences derived from the various parent proteins [40].

*ELISA*

Chagatest recombinante v3.0 / v4.0 (https://www.wiener-lab.com.ar/en-AR/catalog/?sp=chagas) utilises recombinant antigens SAPA, Ag1, -2, -13, -30, and -36 immobilised in the reaction well. According to manufacturer literature, version 3.0 uses a polyclonal anti-human IgG conjugate; version 4.0 uses a monoclonal antibody.

BioELISA Chagas (https://www.werfen.com/oem/bioelisa-chagas) utilises recombinant antigen TcF, a fusion of TcD, TcE, Ag2, and TcLo1 first described in [36].

Abbott Prism Chagas (https://www.fda.gov/media/82145/download) uses recombinant proteins TcF, and FP3, FP6 and FP10; the FP antigens are described as comprised of 2 domains each: FP3 (TcR27 and FCaBP); FP6 (TcR39 and FRA); FP10 (SAPA and MAP) [49]. This system uses the same antigens as the ARCHITECT Chagas assay [50].

MultiCruzi (https://www.infynity-biomarkers.com) uses several known antigenic sequences printed synthetically on the surface of a 96-well plate; sequences recognised by sera are identified by software pixel detection [51].

Thus, as described above and in Table 3, across the differing formats and combinations of individual antigens utilised per test, there is a core group of widely used antigens, principally deriving from the seminal publications of Ibañez and colleagues [16,17].

Commercial assays for serological diagnosis of Chagas disease that are based on antigens without defined sequence, for example, cell lysate, include: Chagatek ELISA (https://www.lab-lemos.com/productos); Chagatest Lisado (https://www.wiener-lab.com.ar/en-AR/catalog/?sp=chagas); Hemagen Chagas Kit (https://www.hemagen.com/product_inserts/66101_06_Chagas_EIA_liquid.pdf); Ortho *T*. *cruzi* ELISA Test System (https://www.fda.gov/media/77498/download).

**Table 3:** Application of defined antigens in commonly reported commercialised tests.

Antigen nomenclature		Application (manufacturer antigen nomenclature)						
Original	Re-identification	RDT			ELISA			
		Chagas Stat-Pak	BIOLINE Chagas Ab	Chagas Detect Plus	Chagatest recombinante	BioELISA Chagas	Abbott Prism	MultiCruzi
**1F8**	FCaBP	1F8	1F8	-	-	-	FCaBP	-
**KMP-11**	-	-	-	KMP-11	-	-	-	KMP-11
**Ag1**	FRA; JL7; H49; Pep1	JL7/H49	H49	Pep1	Ag1	-	FRA	-
**Ag2**	TCR39; B13; Pep2	B13	-	Pep2	Ag2	Pep2	TcR39	Ag2 and TCR39
**Ag7**	SAPA	-	-	SAPA	SAPA	-	SAPA	SAPA and TS^a^
**Ag13**	TcD	-	-	TcD	Ag13	TcD	-	TcD
**Ag30**	TCR27; CRA; Pep30	-	-	Pep30	Ag30	-	TcR27	CRA
**Ag36**	JL9; MAP; Pep36	-	-	Pep36	Ag36	-	MAP	MAP
**TcLo1.2**	-	-	-	TcLo1.2	-	TcLo1.2	-	-
**TCR69**	Tc40; TcE	-	-	TcE	-	TcE	-	TCR69; Tc40; L19^b^

^a^ Trans-sialidase.

^b^ 60S ribosomal protein L19 contains the APAKAA repeat epitope also found in TCR69, Tc40 and TcE.

## Geographical variation in test sensitivity

Endemic Chagas disease has an immense geographical range, from the southern USA to Argentina, with disparate ecological distribution of *T*. *cruzi* DTUs; however, antigen discovery had been largely achieved using sera originating from the “Southern Cone” of South America where TcII, TcV, and TcVI predominate in human infection. In contrast, TcI and TcIV are more prevalent in northern South America and North America [52]. During the last 2 decades, there have been several reports of heterogenous sensitivities of commercial tests across the Americas according to the geographical origin of the samples assayed (Table 4). A common theme of these reports has been that, broadly speaking, sensitivity is highest in the Southern cone of South America, and progressively decreases through northern South America, Central America, and Mexico [43,45,53–59]. The reports’ authors speculated that the inter-regional differences in sensitivities could be due to the geographical variability of the predominant *T*. *cruzi* lineages (DTUs) across the Americas, leading to diversity in certain crucial antigen sequences; an analysis of genomic data across lineages identified <95% sequence conservation in SAPA, Ag30, H49, and B13 [60].

Reports from Mexico [61,62] found that commercial tests were much less sensitive than an ELISA based on a mix of lysates of locally isolated strains. The host immune response stimulated by different lineages may also vary [54,59].

The geographical variations in test sensitivity have a circumstantial similarity to the observed distribution of chronic symptoms across the endemic area, namely gastrointestinal complications (megasyndromes) being much more reported from the “Southern Cone” compared to widespread chagasic cardiomyopathy [5,52]; however, the authors of the reports in Table 4 have not postulated a link.

Conversely, other reports have not observed lower sensitivities across geographical regions using commercial tests or versus local crude extract, including studies on samples from Central America [63] and Mexico [64,65]; another report found no statistically significant differences in sera of donors from Mexico, Central America, and South America when using the novel Chagas Detect Fast ELISA (Inbios) [66].

**Table 4:** Reports of varying geographical sensitivity of Chagas disease serological tests (2008–2021).

Year	Reference	Sample origins^a^	Assays^b^	Comparison/referencec	Authors’ reports
2008	Sosa-Estani et al. [53]	Pregnant women: ARG; BOL; HON; MEX.	RDT: CSP (umbilical cord blood)	ELISA: Chagatest recombinant, v3.0 (maternal blood)	% Seropositive ELISA / RDT: BOL 28.8 / 29.4 ARG 6.6 / 6.2 HON 4.4 / 6.6; MEX 0.8 / 0.9.
2009	Verani et al. [45]	BOL (obstetrical service); PER (mostly public schools)	RDT: CSP; TD.	ELISA: Chagatek (epimastigote lysate)^c^; Chagatest Recombinante. In-house: IFA; RIPA.	% Seropositive TD: BOL 90.7; PER ~54^d^. CSP: BOL 87.5; PER ~30^e^. Chagatek among confirmed positives significantly higher in BOL.
2014	Martin et al. [54]	Arequipa, PER; Santa Cruz, BOL.	ELISPOT: IFNγ release by T cells stimulated by *T*. *cruzi* lysate.	ELISA: Chagatek^c^; Chagatest Recombinante. In-house: IFA; TESA blot; ELISA (using a local *T*. *cruzi* antigen with PER samples).	Chagatek: seropositive value distribution significantly lower in PER. ELISPOT (seropositives): BOL higher median T cell production of IFNγ and response rates.
2018	Buekens et al. [55]	Women enrolled at delivery: ARG, HON, MEX	RDT (cord blood): CSP; TD.	ELISA: Chagatest recombinant, v3.0 (maternal blood).	% of total seropositive cord blood (both RDTs / CSP only / TD only): ARG 83.2 / 14.2 / 2.6; HON 79.2 / 16.9 / 3.9; MEX 34.9 / 33.0 / 32.1. % seropositive maternal ELISA (if ≥1 cord blood RDT positive): ARG 87.0; HON 78.7; MEX 29.4.
2019	Whitman et al. [56]	Blood donors originating from MEX, Central America, or South America	RDT: CDP. ELISA: Ortho *T*. *cruzi*^c^; Hemagen Chagas kit^c^; Chagatest Recombinante v.3.0.	(i) Immunoassays or RIPA used for American Red Cross blood donations (BD); (ii) consensus from this study; (iii) latent class analysis (modelling).	Compared to BD or consensus status, sensitivity for Ortho *T*. *cruzi*, Chagatest Recombinante, and Hemagen tended to lowest in MEX-born, intermediate in Central Americans and highest in South Americans; trend consistent for seropositives by the 4 assays.
2021	Castro-Sesquen et al. [57]	Areas of origin: “TcI” (MEX, Central Americans, PER, CoR, VEN) or “TcII/V/VI” (ARG, BOL, BRA, CHI).	RDT: CDP. ELISA: Hemagen Chagas Kit^c^; Chagatest recombinant v.3.0.	Latent class analysis (modelling). In-house: IgG-TESA-blot using Y strain.	In the latent class with low antibody level: 65.0% of seropositives from TcI- vs. 22.8% from TcII/V/VI-predominant areas.
2021	Castro-Sesquen et al. [43]	Areas of origin “TcI” (MEX, Central Americans) or “TcII/V/VI” (BOL).	RDT: CSP; CDP	Latent class analysis using area of origin and Hemagen Chagas kit^c^, Chagatest recombinante v 3.0, and in-house IgG-TESA-blot.	CSP sensitivity 75.0% in TcI- and 100.0% in TcII/V/VI-predominant areas of origin.
2021	Kelly et al. [58]	Blood donors: born MEX, Central America, or South America.	RDT: CDP. ELISA: Ortho *T*. *cruzi*^c^; Hemagen Chagas kit^c^; Chagatest Recombinante v.3.0 and v4.0; Chagatest Lisado^c^; Abbott PRISM assay.	American Red Cross donor algorithms in use at time of blood donation.	Chagatest Recombinante v4.0, Chagatest Lisado, and Abbott PRISM assay had highest, intermediate, and lowest sensitivity with South American-, Central American-, and MEX-born, respectively, consistent with signal/cut-off ratios.
2021	Truyens et al. [59]	Maternal cord blood: ARG, HON, MEX.	RDT: CSP; TD. ELISA: Chagatest ELISA recombinant v3.0	-	% Seropositives of CSP / TD / ELISA: ARG 97.3 / 86.6 / 87.9; HON 96.1 / 82.9 / 78.5; MEX 67.3 / 66.4 / 27.9. Consistent with combinations of tests, and with ELISA antibody levels.

^a^ARG Argentina; BOL Bolivia; BRA Brazil; CHI Chile; CoR Costa Rica; HON Honduras; MEX Mexico; PER Peru; VEN Venezuela.

^b^RDTs: CDP Chagas Detect Plus; CSP Chagas StatPak; TD Trypanosoma Detect.

^c^Commercial ELISA kits using undefined native *T*. *cruzi* lysate antigens (see text): Chagatek ELISA; Chagatest Lisado; Hemagen Chagas Kit; Ortho *T*. *cruzi* ELISA Test System.

^d^Authors’ report: Observer 1, 26.6%; Observer 2, 33.0%.

^e^Authors’ report: Observer 1, 53.8%; Observer 2, 54.6%.

IFA, indirect immunofluorescence assay; RIPA, radioimmunoprecipitation assay; TESA, trypomastigote excretory-secretory antigen.

## Opportunities

Areas which present opportunities for future serological development include monitoring treatment outcome, congenital infection, and lineage-specific serological antigens.

*Treatment monitoring*. IgG serology may remain positive for many years following treatment, confounding test-of-cure. Several antigens have been proposed as serological biomarkers, measured as decrease in specific reactivity after anti-trypanosomal drug treatment of patients. These include defined antigens used in commercialised tests: F29 (1F8/FCaBP) [67]; KMP11, paraflagellar rod protein-2, Heat shock protein 70 and peptide 3973 (from CA-2) [68]; MAP [69]; TSSA-VI [70]; TcTASV antigens [71]; Tc_5171 [72]. An expert consensus describing the target product profile requirements for early assessment of treatment efficacy has been published recently [73]. As an alternative approach, in a study of Argentine patients following chemotherapy of early chronic Chagas disease, a significant decline in IgG1 suggested cure, whereas sustained or increased IgG1 was an indicator of treatment failure [74].

*Congenital*. Serology of neonates (to 9 months) is complicated by the persistence of maternal IgG, so detection of the infant’s IgM may be a useful alternative [75]. Truncated or fusion forms of SAPA have been reported for detecting IgM [76,77]. In the TESA (trypomastigote excretory-secretory antigen) western blot format, the presence of characteristic bands (130 to 200 kDa size range) have been used for diagnosis [78–80]. An association between maternal IgG1 and IgG2 levels and congenital transmission has been reported [81]. Another development has been the Chunap assay using nanoparticles for antigen detection in urine [82].

*Lineage-specific*. As described above, the original classification into DTUs, or genetic lineages, was based on non-antigen coding sequences. Commercial tests are not designed to identify antibodies made to specific *T*. *cruzi* lineages; identification of lineage-specific serological antigens has the potential to reveal broader knowledge of an individual’s history of lineage infection, and may elucidate associations with clinical presentation or ecological cycles. The only robust such antigen to be identified is the trypomastigote small surface antigen (TSSA) [83,84]. The TSSA-II/V/VI shared epitope particularly has been applied to sera from humans and. An association between anti-TSSA-II/V/VI IgG and severity of cardiac pathology in chronic chagasic patients has been reported [87]. Although not widely exploited in commercial tests, it has been incorporated into multiepitope fusion proteins with MAP, TcD, FRA, and SAPA [77]. The application of TSSA-II/V/VI serology also has potential to elucidate ecological cycles of mammalian host species such as dogs and primates [85,86], and, with a wider range of lineage-specific antigens, to a greater range of silvatic reservoirs [88].

*Recent approaches*. The application of fine epitope mapping to the *T*. *cruzi* proteome [89] and TSSA-II [90] seeks to define crucial epitopes; their application to future refinement of serological tests presents a novel prospect.

The issue of serological diagnosis and management of Chagas disease in immunocompromised patients has been reviewed recently elsewhere [91].

### Conclusions

The advent of molecular biology techniques empowered pioneering researchers to identify defined antigens, several years before the advent of published *T*. *cruzi* genomes or, in some cases, of automated DNA sequencing. Their enduring legacy is that decades later these antigens and epitopes continue to be incorporated in today’s commercial tests. Standardised production has allowed wider application and deployment, particularly as RDTs in settings where more sophisticated research facilities are not available.

Research gaps provide opportunities for future refinement, in terms of parasite genetic diversity (TcI-TcVI), geographical sensitivities, treatment outcome monitoring, congenital transmission, the utility of assaying IgG subclasses, and the relevance of lineage-specific serology in human disease and mammalian infection. Next-generation sequencing technology facilitates the genomes of an ever-expanding range of *T*. *cruzi* isolates to be made available online, and mapping of candidate antigens can allow refinement of epitopes; these resources, unavailable to the early researchers, will surely guide and expedite future development, including fine-tuning to specific epidemiological situations. This is of crucial importance in improving healthcare services to populations that are currently underserved or poorly recognised. The history given herein can also serve as a guide for other infectious diseases.
